# White gastric mucosa during endoscopy as a new endoscopic feature of chronic ischemic gastritis: A case report

**DOI:** 10.1002/deo2.192

**Published:** 2022-12-13

**Authors:** Chikamasa Ichita, Akiko Sasaki, Naoko Isogai, Chihiro Sumida, Takashi Nishino, Jun Kubota, Kento Shionoya, Karen Kimura

**Affiliations:** ^1^ Gastroenterology Medicine Center Shonan Kamakura General Hospital Kanagawa Japan; ^2^ Department of Surgery Shonan Kamakura General Hospital Kanagawa Japan; ^3^ Department of Gastroenterology and Hepatology Tokyo Medical University Tokyo Japan

**Keywords:** celiac artery stenosis, chronic ischemic gastritis, gastric ulcers, superior mesenteric artery occlusion, surgical bypass

## Abstract

Chronic ischemic gastritis (CIG) requires early diagnosis and treatment as complications of thromboembolism can be fatal. Although computed tomography (CT) is useful in the diagnosis of CIG, it is difficult to diagnose from a patient's history, endoscopic findings, and tissue biopsy. Identification of the key findings that motivate computed tomography is an important issue. We report a case of CIG diagnosed by endoscopic findings of white patches of mucosa over time. A 63‐year‐old man presented with epigastric pain. He had a history of repeated gastric ulcers of an undetermined cause. We performed upper endoscopy and observed the appearance of multiple white patches on the gastric mucosa. Central vessel stenosis was considered, and aortic computed tomography revealed complete occlusion of the superior mesenteric artery and stenosis of the celiac artery. We carried out a surgical bypass and found no postoperative endoscopic mucosal changes or abdominal pain. White patch changes in the gastric mucosa over time during endoscopy may indicate CIG. This finding may help in the future diagnosis of CIG.

## INTRODUCTION

Chronic ischemic gastritis (CIG) is a rare disease, with only 26 cumulative cases reported over the past 40 years. The diagnosis is difficult based on clinical symptoms, which include chronic abdominal pain that worsens with food, weight loss over several months, vomiting, gastric paresis, diarrhea, and constipation.^1^, ^2^, ^3^, ^4^, ^5^, ^6^, ^7^, ^8^, ^9^, ^10^ Endoscopic findings are nonspecific, including ulceration, erosion, and gastritis,^1^, ^2^, ^3^, ^4^, ^5^, ^6^, ^7^, ^8^, ^9^, ^10^ and histological diagnosis by biopsy is also difficult.^7^ Computed tomography (CT) is considered the most useful examination tool for definitive diagnosis.^9^ However, the diagnosis of CIG is difficult as definitive findings are not possible on CT.^5^, ^9^ The best treatment is revascularization, usually surgical, although a percutaneous approach may be used in high‐risk cases.^5^, ^6^, ^8^, ^9^ The importance of early diagnosis is emphasized by the fact that the prognosis may be poor if therapeutic intervention is not undertaken.^9^ Although, the diagnosis of CIG is difficult, a missed diagnosis can be fatal; therefore, the identification of new findings that may help make the diagnosis easier is a crucial issue.

We report a case of CIG, including a video clip, diagnosed after upper endoscopy revealed patchy white changes in the gastric mucosa, and a background blood flow disorder was detected. Although various endoscopic findings of CIG have been reported in the past, there have been no reports focusing on changes in mucosal coloration over time, particularly on video. This finding may help in the future diagnosis of CIG.

## CASE REPORT

**FIGURE 1:**
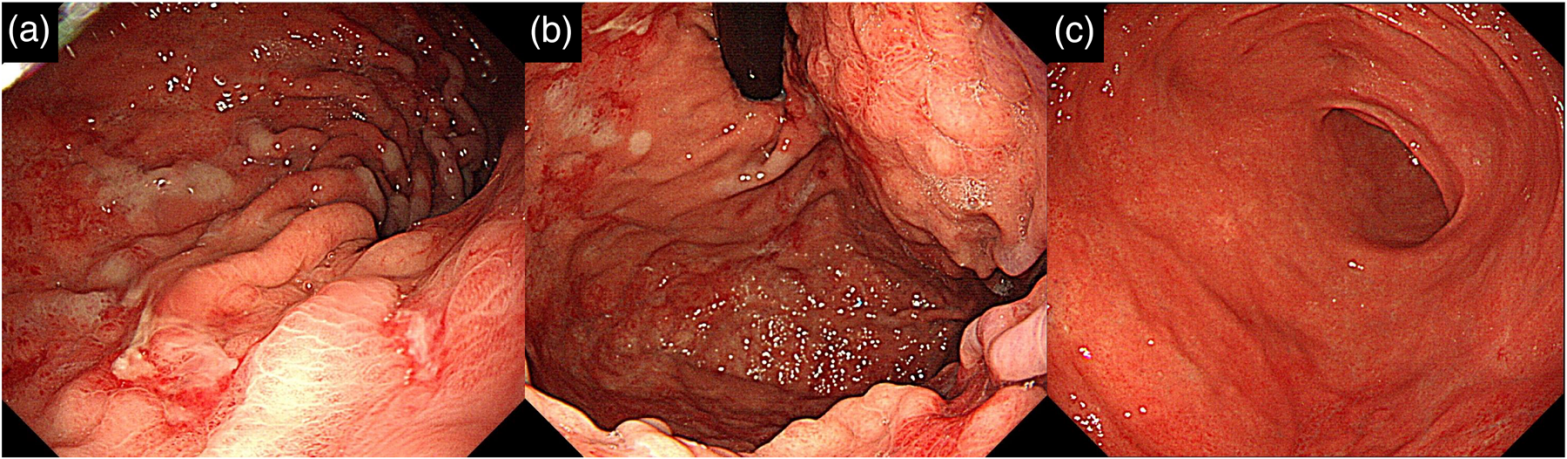
Upper endoscopic images at the patient's initial presentation. GIF‐H260 was used for observation. (a) The posterior wall of the upper stomach body. (b) Fundus. (c) Antrum. Multiple erosions and shallow ulcers were seen on the posterior wall from the stomach body to the fundus. A white mucosal change was also observed around the ulcer. No findings were seen in the antrum

A 63‐year‐old man presented for investigation due to recurrent gastric ulcers. Five years prior, an upper endoscopy was performed to investigate the cause of his persistent severe epigastric pain, which revealed multiple erosions and ulcers in the stomach (Figure 1). The patient had hypertension and hyperlipidemia, but no diabetes or chronic kidney disease. He was negative for *Heliobacter pylori* antibodies and had no evidence of previous eradication. He also did not use nonsteroidal anti‐inflammatory drugs or anti‐thrombotics. The biopsy results showed only inflammatory findings. The cause of the ulcer was unclear, and the gastric mucosa improved with the administration of proton pump inhibitors. The patient developed repeated gastric ulcers; however, the mucosal damage was not as severe as at the initial presentation.

**FIGURE 2:**
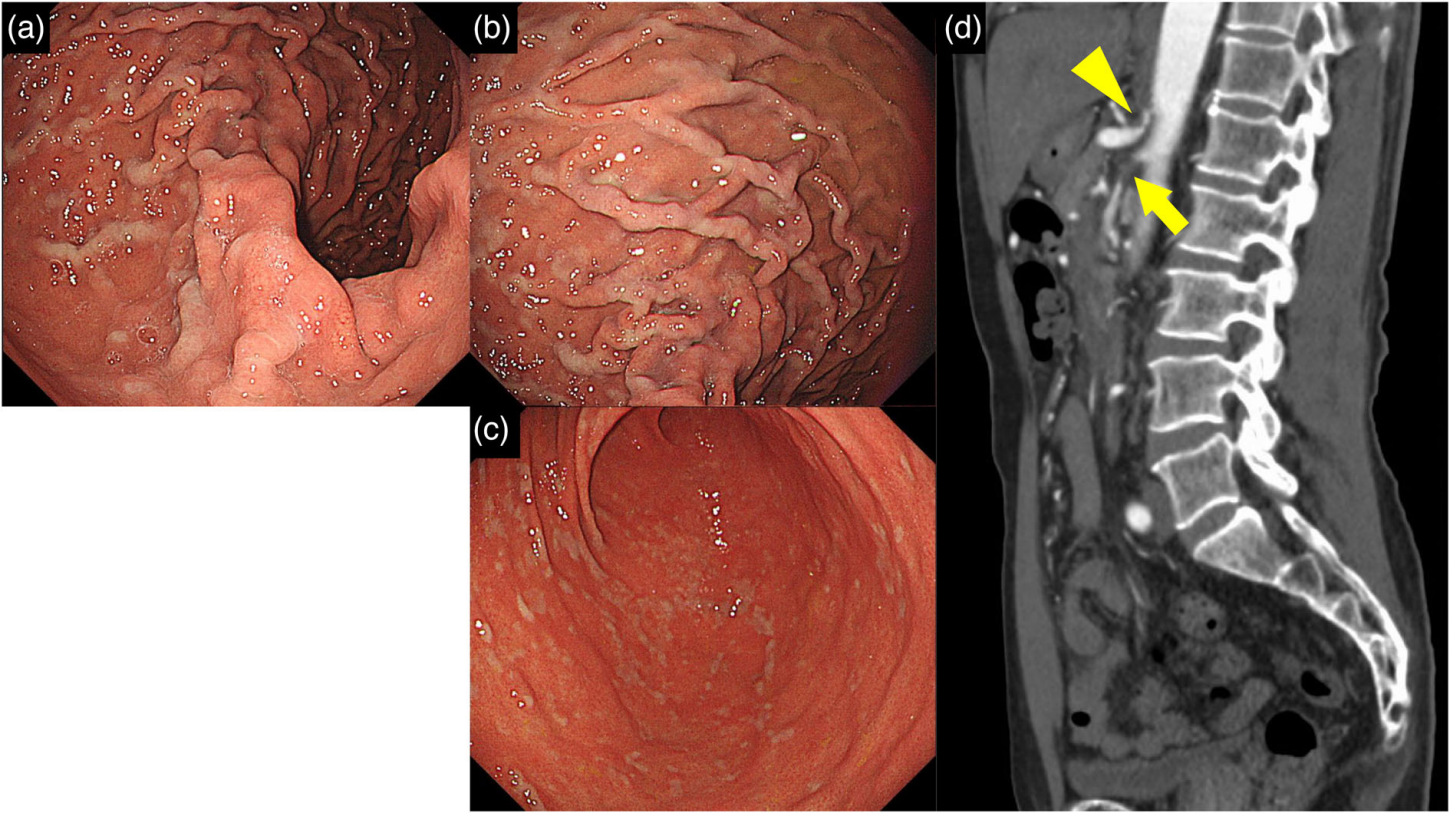
Endoscopic and Angiographic computed tomography findings at diagnosis. GIF‐H290Z was used for observation. (a) The posterior wall of the upper stomach body. (b) Fundus. (c) Antrum. Endoscopic findings after gastric dilatation. The mucosa was white in patches. (d) Angiographic computed tomography images. The superior mesenteric artery was occluded but was nourished by collateral circulation (arrow). The celiac artery was seen to be narrowed and dilated its periphery (arrowhead)

In the present case, the patient has similar epigastric pain, and he was introduced to our hospital to investigate the cause of the pain. We performed upper endoscopy and found that the gastric mucosa became white over time due to gastric dilatation by endoscopic air insufflation (Video S1; Figure 2a–c). Tissue biopsy from white mucosa showed no specific findings.

**FIGURE 3:**
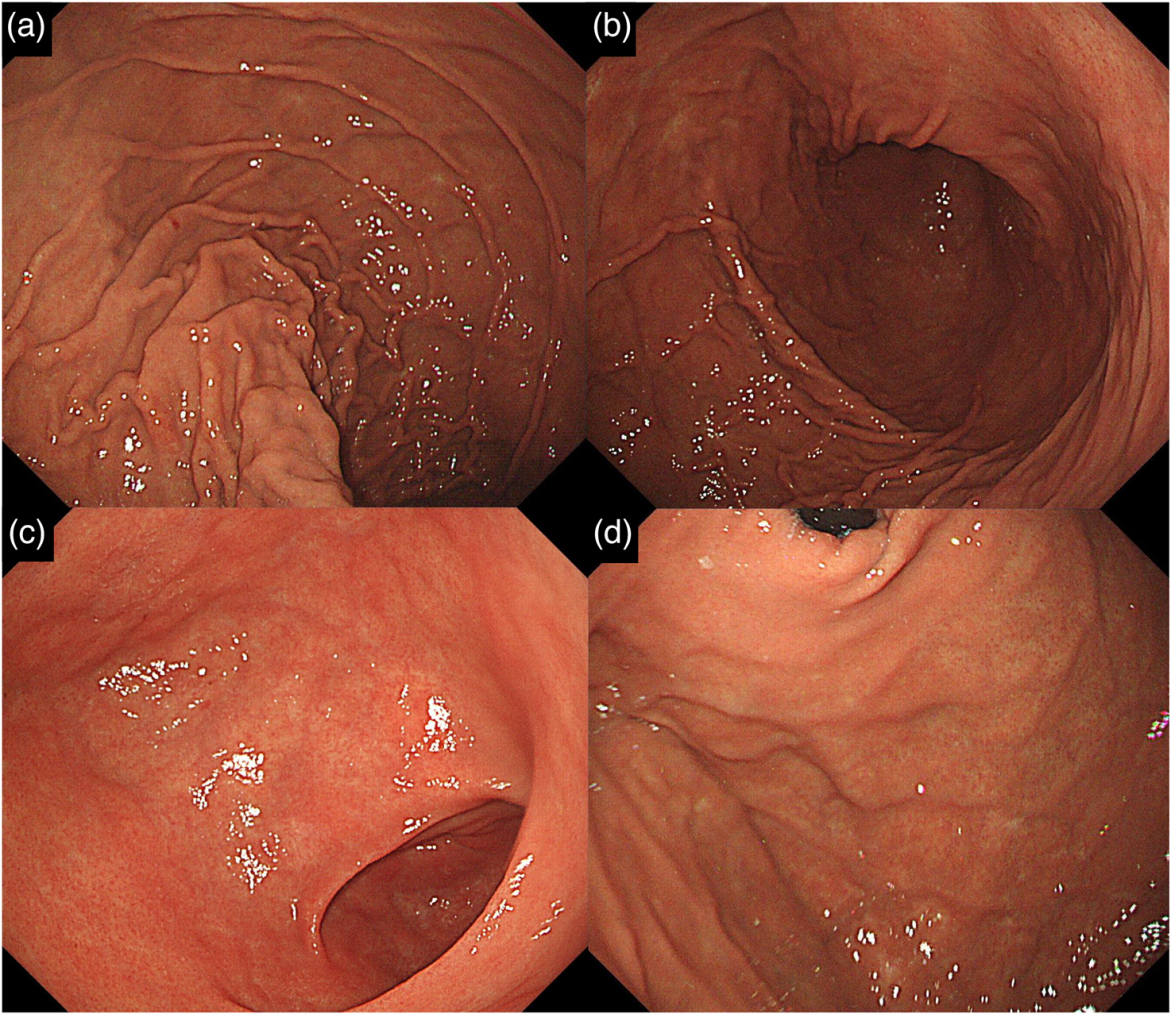
Upper endoscopic images after bypass surgery. GIF‐H290Z was used for observation. (a) The posterior wall of the upper stomach body. (b) Stomach body. (c) Antrum. (d) Fundus. No change in the color of the mucosa was observed, even when the stomach was sufficiently dilated

We considered the possibility of ischemic changes in the perigastric vessels and performed aortic CT on the same day. The results indicated complete occlusion of the superior mesenteric artery (SMA) and stenosis of the celiac artery (Figure 2d). No obvious cause of stenosis and occlusion was found, and atherosclerosis was determined to be the cause. At this point, the patient had no obvious evidence of organ ischemia. Blood flow to the intra‐abdominal organs was maintained by blood flow from the inferior mesenteric artery and a small amount of blood flow from the SMA. The only current symptom was abdominal pain after eating, and the patient was followed up as an outpatient. We considered that (1) the patient still had recurrent abdominal pain and (2) if thromboembolism occurred in the inferior mesenteric artery in the future, the patient would be at risk of developing intra‐abdominal organ ischemia, which would be life‐threatening. Therefore, after consultation with the patient, we performed a surgical bypass two weeks after diagnosis. A bypass was created between the inferior mesenteric artery, the SMA, and the common hepatic artery using the right great saphenous vein. Postoperative endoscopy indicated no color changes, even with gastric dilatation (Figure 3). Postoperatively, the patient no longer had abdominal pain.

## DISCUSSION

Here, we report a case of CIG diagnosed during upper endoscopy with a change over time in mucosal color with dilation of the gastric wall. This finding enabled early detection of central vascular obstruction and avoided a fatal outcome.

CIG is a rare disease, reported in a limited number of cases, and no clear diagnostic criteria have been established.^1^, ^2^, ^3^, ^4^, ^5^, ^6^, ^7^, ^8^, ^9^, ^10^ In previous reports, CIG was diagnosed when gastric mucosal injury with stenosis or obstruction of the celiac artery or mesenteric artery.^1^, ^2^, ^3^, ^4^, ^5^, ^6^, ^7^, ^8^, ^9^, ^10^ Endoscopic findings of CIG have been reported as multiple white irregular ulcers surrounded by mottled erythema, which is similar to the initial endoscopic findings in this case. Therefore, the present case was diagnosed as CIG.

The possible causes of the change in mucosal coloration were the following: (1) complete occlusion of the SMA or (2) stenosis of the celiac artery resulting in insufficient blood flow to the stomach. Furthermore, (3) the stretching of the gastric wall by endoscopic insufflation may have further stretched and compressed the internal vessels of the stomach, resulting in a decrease in blood flow. The possibility of (1) and (2) is plausibly supported because these mucosal changes were no longer observed after the surgical bypass. Hypothesis (3) was assumed because the stomach was dilated by insufflation and mucosal changes were observed, although no white color changes were observed in the collapsed gastric mucosa. Further, once the stomach was fully dilated, no white mucosal development was observed. However, there is no previous literature to explain these findings, and further case series and animal experiments are needed to prove this hypothesis.

Previous reports have shown that diagnosis is difficult to make based on endoscopic and histologic findings.^1^, ^2^, ^3^, ^4^, ^5^, ^6^, ^7^, ^8^, ^9^, ^10^ Endoscopic findings include multiple ulcers and erosions, as shown in Table 1, but other findings, such as gastritis, have been noted, and there are no definitive findings. The tissue results from the ulcer at the initial endoscopy only showed inflammatory findings, and the tissue results from the white mucosa did not show any specific findings. Once an erosive ulcer is formed, ischemic changes are difficult to detect due to inflammatory cell infiltration, and temporary white color changes alone do not indicate mucosal changes. As reported in previous cases, the diagnosis of CIG in biopsy tissue is considered difficult.^7^

**TABLE 1:** Reported cases of chronic ischemic gastritis

**First author**	**Year**	**Cases**	**Endoscopic findings**	**Diagnosis by biopsy**	**Treatment**	**Outcome**
Force	1980	1	Multiple superficial ulcerations, irregular areas of raised mucosa, and atrophic appearance	No	No	Expired
Allende	1982	1	Multiple small mucosal erosions, small ulcer at the gastric antrum, and duodenal hyperemia	No	No	Expired
Cherry^1^	1986	4	Multiple and shallow ulcers with sloping edges and irregular shapes	No	1. Percutaneous balloon dilation; 2. Surgery; 3. Percutaneous treatment failure, successful surgery; 4. Percutaneous treatment failure, successful surgery	All alive
Hojgaard^2^	1987	1	Multiple small acute ulcerations, one chronic pyloric ulcer, and duodenitis	No information	Surgery	Alive
Bouche^3^	1989	1	Multiple non‐hemorrhagic antral erosions with irregular outlines and a pseudomembranous floor	No	Surgery	Alive
Liberski^4^	1990	2	Superficial antral ulceration and mild superficial gastric and pyloric ulcerations	No information	Surgery	Alive
Babu^5^	1993	6	Diffuse superficial microulceration in the stomach with pale mucosa	No information	5/6 surgery; 1 no	5 expired; 1 alive
Casey^6^	1993	7	Gastritis (various sites and degrees)	No	6/7 surgery; 1 no	Treated, 4 alive, 2 expired; Untreated, expired
Trowell^7^	1998	1	Severe gastritis and duodenitis, and multiple ulcers	Yes	no	Expired
Haberer^8^	2003	1	Markedly abnormal gastric mucosa with multiple malignant appearing ulcerations	No	Surgery	Alive
Quentin^9^	2006	1	Ulceration in the body of the stomach, along the posterior aspect of the greater curvature. Regenerative phenomena and an atrophic area are visible in the fundus. Retrograde view of necrotic ulceration along the posterior aspect of the greater curvature	No	Percutaneous treatment failure	Expired
Daher^10^	2016	1	Upper Endoscopy showed multiple gastric ulcers in the body of the stomach, fundus, and pylorus	No	Surgery	Alive

Surgical bypass is the most common treatment for CIG.^1^, ^5^, ^6^, ^9^ On the other hand, patients in poor general condition are treated with percutaneous intervention, but the prognosis for percutaneous intervention is inferior to surgery.^3^, ^8^, ^9^ In the present case, as CIG was detected at an early stage and the patient was in good general condition, a surgical vascular bypass was suitable. Diagnosis by a mucosal color change over time may be effective for the early detection of CIG.

We experienced a case in which mucosal changes were identified over time during upper endoscopy, enabling early diagnosis of CIG. When endoscopic patchy white changes of the gastric mucosa are observed, the vascular disease should be suspected, and aortic CT or angiography should be recommended.

## CONFLICT OF INTEREST

None.

## Supporting information




**Video S1** Video of gastric mucosal changes
